# Uncovering Viral Protein-Protein Interactions and their Role in Arenavirus Life Cycle

**DOI:** 10.3390/v4091651

**Published:** 2012-09-21

**Authors:** Maria Eugenia Loureiro, Alejandra D’Antuono, Jesica M. Levingston Macleod, Nora López

**Affiliations:** 1 Centro de Virología Animal (CEVAN), Instituto de Ciencia y Tecnología Dr. Cesar Milstein, Consejo Nacional de Ciencia y Tecnología (CONICET), Saladillo 2468, Buenos Aires C1440FFX, Argentina. Email: eugenialoureiro@yahoo.com.ar (M.E.L.); adantuono@gmail.com (A.D.A.); 2 Department of Microbiology, Mount Sinai School of Medicine, One Gustave L. Levy Place, New York, NY 10029, USA. Email: jesica.levingstonmacleod@mssm.edu (J.M.L.M.)

**Keywords:** Tacaribe virus, protein-protein interaction, viral RNA synthesis, virus assembly

## Abstract

The *Arenaviridae* family includes widely distributed pathogens that cause severe hemorrhagic fever in humans. Replication and packaging of their single-stranded RNA genome involve RNA recognition by viral proteins and a number of key protein-protein interactions. Viral RNA synthesis is directed by the virus-encoded RNA dependent-RNA polymerase (L protein) and requires viral RNA encapsidation by the Nucleoprotein. In addition to the role that the interaction between L and the Nucleoprotein may have in the replication process, polymerase activity appears to be modulated by the association between L and the small multifunctional Z protein. Z is also a structural component of the virions that plays an essential role in viral morphogenesis. Indeed, interaction of the Z protein with the Nucleoprotein is critical for genome packaging. Furthermore, current evidence suggests that binding between Z and the viral envelope glycoprotein complex is required for virion infectivity, and that Z homo-oligomerization is an essential step for particle assembly and budding. Efforts to understand the molecular basis of arenavirus life cycle have revealed important details on these viral protein-protein interactions that will be reviewed in this article.

## 1. Introduction

Arenaviruses are phylogenetically divided into the Old World (OW) and the New World (NW) groups, both of which include human pathogens associated with hemorrhagic fever, an often fatal disease [1,2]. Representative of the OW group are Lassa fever virus (LASV), which causes a high number of hemorrhagic fever cases in West Africa, and the worldwide-distributed Lymphocytic Choriomeningitis Virus (LCMV), a causative agent of aseptic meningitis. The NW group is comprised of all known South American pathogens that produce severe hemorrhagic disease: Junin (JUNV), associated with a major health problem in rural areas of Argentina, Machupo (MACV), Chapare, Guanarito, and Sabia viruses. This group also includes the prototypic, non-pathogenic Tacaribe virus (TCRV) [3]. TCRV is genetically and antigenically closely related to JUNV [3,4] and has been largely used as experimental model in our studies on NW arenavirus molecular genetics.

TCRV, like all arenaviruses, is an enveloped virus whose genome consists of two single-stranded, negative-sense RNA segments named S and L. Two genes, arranged in opposite orientation and separated by a non-coding intergenic region, are comprised in each genomic segment. The S RNA encodes the Nucleoprotein (N, 64 kDa) and the glycoprotein precursor (GPC, ca. 70 kDa). The L segment encodes the viral RNA-dependent RNA polymerase (L protein, 240 kDa) and a small protein called Z (11 kDa) [5]. The coding sequences are expressed from subgenomic messenger RNAs (mRNAs) transcribed from the 3´ regions of the genomes and antigenomes. These mRNAs are non-encapsidated and non-polyadenylated at their 3’ ends. Their 5’ ends contain short stretches of additional bases that are capped, which suggests that arenaviruses (like influenza viruses and bunyaviruses) may use a “cap-snatching” mechanism to initiate mRNA synthesis [5,6,7]. Mapping of the 3’ ends of TCRV mRNAs within the corresponding RNA intergenic region, along with our studies on the requirements for viral mRNA synthesis indicate that transcription termination is associated with sequence-independent signals located within the intergenic regions. These signals consist of -at least- a stable single hairpin structure that is not required for a correct initiation of transcription or replication [8,9]. 

In addition to the intergenic region, all arenaviruses genomic segments contain 5’ and 3’ terminal non-coding sequences that display inverted complementarity and are predicted to form panhandle structures, which harbor the promoter signals for viral RNAs synthesis [10,11,12]. 

## 2. Protein-protein interactions in viral RNA synthesis.

### 2.1. Z protein inhibits viral RNA synthesis by direct interaction with the L polymerase.

**Figure 1:**
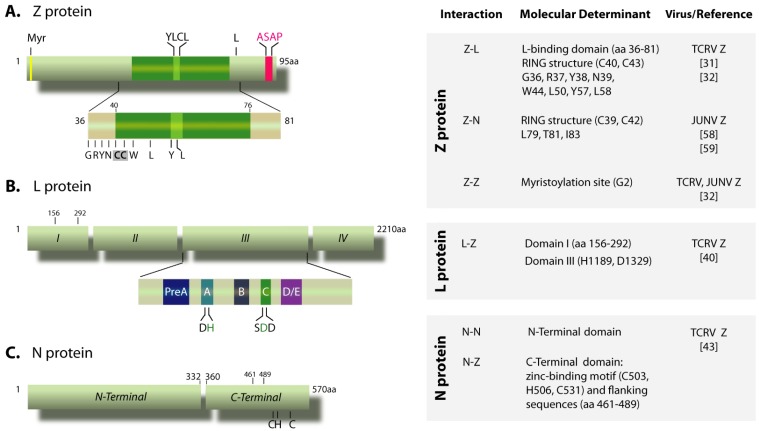
**Schematic representation of Tacaribe virus (TCRV) N, L and Z proteins**. **(A)** Z protein. The myristoylation site at the G2 residue (Myr), the YLCL and ASAP motifs and the conserved L residue at position 80 (equivalent to L79 in JUNV Z) are indicated on top. The insert represents the L-binding domain (residues 36-81), showing amino acid residues critically important for binding the L protein. The first two cysteine residues (C40, C43) of the RING motif are shadowed. Numbers on top correspond to the positions limiting the RING domain (indicated by a green box). **(B) **L protein. The conserved domains I to IV and motifs pre-A to E within domain III (insert) are indicated. Numbers on top correspond to the positions limiting the N-terminal region required for the interaction with Z. The conserved H and D residues within motifs A and C that are involved in binding Z are shown in green. **(C) ** Scheme of the N protein, showing the N-terminal domain (amino acids 1 to 332) involved in self-association, and the C-terminal domain (amino acids 360 to 570) engaged in the interaction with Z protein. The indicated cysteine and histidine residues, which conform a conserved zinc-binding motif [45,76], as well as the sequence spanning positions 461 to 489, are essential for Z binding. The chart on the right displays the experimental data on the viral protein-protein interactions discussed in the text.

The arenavirus Z gene was the last to be identified once the sequences of TCRV and LCMV L segments were completed [13,14]. Comparison of the deduced Z protein sequences published thus far reveals a similar organization consisting of a conserved central 37-amino acid (aa) RING domain, and less conserved N- and C-terminal domains (Figure 1A). The RING (Really Interesting New Gene) domain is defined by a conserved pattern of cysteine and histidine residues (C1-X_2_-C2-X_9_-C3-X_2_-H1-X_2_-C4-X_2_-C5-X_10_-C6-X_2_-C7, where X can be any aa) that coordinate two zinc ions in a cross-brace arrangement, an essential requirement for domain folding [15]. A characteristic feature of RING domains is their ability to mediate protein-protein interactions. Indeed, Z has been shown to associate with a number of cellular proteins such as the translation initiation factor eIF4E, promyelocytic leukemia (PML) protein, ribosomal P proteins and proline-rich homeodomain protein, as well as retinoic acid inducible gene I (RIG-I). These interactions have been proposed to modulate host cells growth, apoptosis and induction of antiviral response [16,17,18,19,20,21,22,23]. Despite some RING-containing proteins can act as E3 ligases in ubiquitin-dependent protein degradation, to date no ubiquitin ligating activity has been demonstrated for Z protein [17]. 

The fact that the Z protein displays no sequence homology to any other negative-stranded RNA virus protein, fueled the investigation of its function in viral multiplication. Early studies on LCMV [24]; and TCRV (Lopez, unpublished), indicated that a 400-nt genomic-sense RNA containing the Z gene sequence is packaged into virions. Moreover, the proportion of this small RNA (referred to as Z RNA) to full-length genomic L RNA was found to be lower in cytopathic TCRV stocks than in virus stocks producing non-cytopathic infections, characterized by lower levels of viral RNAs synthesis and viral gene expression. These observations led us to suggest a possible role of Z RNA and∕or Z protein in the outcome of the infection [25]. 

With the establishment of a reconstituted transcription and replication system based on plasmid-supplied viral RNAs and proteins we began to unravel the role of Z protein in TCRV RNA synthesis. Using this system, we demonstrated that L and N are the only viral proteins required for full-cycle viral RNA replication and transcription and that Z protein is a potent inhibitor of both processes [26]. Similar results were obtained from LCMV- [27,28] and, more recently, from a number of OW and NW arenavirus-reconstituted systems [29,30]. 

To understand the mechanism of Z-mediated inhibition of viral RNA synthesis, we investigated possible interactions between TCRV Z and either of the proteins, L or N, essential for these processes. It was determined that Z, at expression levels producing a potent inhibition of transcription and replication, interacted with L but not with N, providing the first evidence for Z-L interaction [31]. Mutagenesis studies on the 95-aa-TCRV Z protein led us to identify a central region (positions 36 to 81) essential for both, L polymerase binding and viral RNA synthesis inhibition. This region includes the entire RING domain plus 4 N-terminal and 5 C-terminal flanking residues. Within this region, both the integrity of the RING structure as well as a number of conserved and non-conserved residues located around the first two cysteine residues of the RING motif (zinc-binding site I) (Figure 1A), were defined to be critically important for TCRV Z binding to L and Z inhibitory function. Taken together, these results led us to postulate that Z interferes with viral RNA synthesis by direct interaction with the L polymerase [31,32]. 

### 2.2. Z protein binds to the putative catalytic site of the L polymerase

Sequence alignments of the L protein of arenaviruses (ca. 2200aa) and RNA-dependent RNA polymerases (RdRps) from a number of negative-stranded RNA viruses first revealed conserved amino acids clustered into four domains (numbered I to IV from the N-terminus) joined by variable regions (Figure 1B). Domain III (ca. 500 aa in length), exhibiting six sequence motifs centered around amino acids invariant in viral RdRp, has been proposed to be part of the catalytic site of the polymerases [33,34,35,36]. The multidomain organization of the arenavirus L protein has been recently supported by electron microscopy images of purified MACV L protein [12], by evidence that LCMV L protein can be split into N- and C-terminal domains that reconstitute a functional polymerase by trans-complementation [37], and by the fact that the N-terminal 196 residues of L (domain I), proposed as a putative endonuclease cap-snatching domain, is able to bind and cleave RNA by itself [38,39].

In order to gain a better understanding of the mechanism of Z-mediated viral RNA synthesis inhibition, our laboratory investigated the Z-protein binding sites on TCRV L protein. Deletion analysis led us to identify two non-contiguous regions as being critical for L-Z interactions. The first region is localized within the N-terminal domain I, between residues 156 and 292, whereas the second one involves domain III (Figure 1B). The C terminal region of L (up to ca. 50 aa downstream from domain III), although essential for polymerase activity, is dispensable for Z binding [40].

The importance of domain III in binding Z was revealed by point mutations in sequence motifs A and C, each of these centered around an aspartic acid invariant in all viral RdRps. Within motif A, we changed either the invariant D1188 or H1189, the latter conserved in all arenavirus L proteins, finding that only H1189 is critically important for binding Z [40]. Motif C exhibits the SDD sequence conserved in segmented negative-stranded virus RdRps, the first D residue of the triplet (D1329) being strictly conserved in all classes of polymerases whereas the second D is somewhat flexible, as it is replaced by N in non-segmented negative-stranded RdRps [33]. Mutational analysis of each residue of the SDD triplet showed that only D1329 is strictly required for Z binding (Figure 1B). Although in all probability other residues in domain III may be involved in interactions with Z, it is a relevant finding that H1189, conserved in all arenavirus L proteins, and the integrity of the SDD motif, present in the core of the polymerase domain and predicted to coordinate a catalytically essential magnesium ion, are critically involved in binding Z [40]. 

The association between L and Z was recently confirmed by Kranzusch *et al.* [30] using an *in vitro* system reconstituted with purified MACV proteins. The authors reported that the Z-L complex locks the viral polymerase in a promoter-bound state that prevents all detectable RNA synthesis. These results are consistent with our data on Z binding to the putative catalytic site of the L polymerase. 

The finding that modifications in either domain III or in the N-terminal region of L impaired the interaction with Z [40] could imply that both regions are required in concert for binding. In this context, it should be considered that arenavirus L protein form oligomeric structures that may engage at least one interaction site at its N-terminal domain [37,41]. It is tempting to speculate that interactions between Z and sequences at the N terminus of L prevent L oligomer formation allowing Z association with the L monomer. Alternatively, modifications in the N terminus of L might affect L oligomerization and, consequently, Z binding as well. Noteworthy, the *in vitro* L-Z complex appears to involve monomeric forms of L and Z [30]. Further, Z mutants unable to self-interact showed unaltered abilities to interact with L and to inhibit TCRV minigenome replication, suggesting that Z monomer represent the functional unit for Z-mediated polymerase activity regulation ([32], and see below).

### 2.3. Other viral protein-protein interactions involved in RNA synthesis

The N protein is the most abundant viral polypeptide in both infected cells and virions, and tightly binds to genomic and antigenomic RNAs to form the viral nucleocapsids. The finding that N is required along with the L protein to promote full-cycle RNA replication of S genome analogs (minigenomes) in several arenavirus-reconstituted systems [26,27,29,42], indicates that the nucleocapsids serve as the functional templates for viral RNA synthesis by the L polymerase. Accordingly, nucleocapsid assembly is thought to be critically important for viral RNA synthesis, and has been largely assumed to depend -at least in part- on the ability of N protein to self-interact. Our studies on TCRV provided the first evidence that arenavirus N protein is able to associate with itself into putative dimeric and trimeric forms within mammalian cells [43]. The putative dimer appeared to be the prevalent form, an observation that is consistent with results obtained for the LCMV N protein [44]. 

The characterization of the ability of TCRV N deletion and point mutants to sustain homotypic (N-N) or heterotypic interactions led us to define two (N-terminal and C-terminal) functional domains within the N protein, and to identify the N-terminal domain (residues 1 to 332) as being responsible for N self-interaction (Figure 1C). Further support for the relevance of the N-terminal domain in self-association has been provided for the LCMV N protein [44]. The atomic structure of LASV N, which was crystallized as trimers in the absence of RNA, confirmed that the LASV N protomer is composed of well-formed N-terminal (residues 7–338) and C-terminal (residues 364–561) domains [45,46]. Comparison of the LASV N RNA-free form structure to that of the N-terminal domain (residues 1–340), crystallized as a complex with non-viral RNA, revealed structural differences suggesting that binding to RNA could be associated with possible conformational changes in N oligomers [47]. Such RNA-induced flexibility of N might explain our and other’s observations pointing to its N-terminal domain as being crucial for self-interaction [43,44], which are in contrast to the predominant head-to-tail (C-terminal-to-N-terminal) tight dimerization interface predicted by the RNA-free protein structure [45,46]. Importantly, mutations introduced in the TCRV N protein that impair protein self-association also abrogate its ability to sustain minigenome RNA synthesis ([43], D’Antuono, unpublished results). Thus, N homotypic interactions may play a significant role in N multimerization on the viral RNA for nucleocapsid formation, and may be required to maintain nucleocapsid architecture during replication, as suggested for other negative-stranded RNA viruses [48,49]. Notably, a conformational switch has been proposed to be at the basis of multimerization and RNA encapsidation by N proteins from the *Bunyaviridae,* another segmented negative-stranded RNA viruses family [50].

Evaluation of other protein-protein interactions in the TCRV reverse genetic system showed that L and N interact with each other [31], an observation supported by recent experimental data for several OW arenaviruses [42]. Importantly, N-L association did not disrupt Z-L complex formation, indicating that the Z and N binding sites on L are independent of each other [31]. Although the precise L and N interacting domains and the specific role of this interaction in the arenavirus life cycle is currently unknown, it is conceivable that N-L interaction might be required for the transient release of the RNA template from the nucleocapsid, as well as for polymerase movement along the template during replication and transcription [47].

## 3. Viral protein-protein interactions in particle assembly

### 3.1. Z-N interaction is important for nucleocapsid packaging

Studies on the OW LASV and LCMV first indicated that Z protein, which is a structural component of the virions, is the main force driving the cell surface budding of arenavirus particles [51,52,53]. Both the integrity of Late domains (PTAP and/or PPPY) at the C-terminus of the protein, which mediate the recruitment of specific cellular proteins of the ESCRT (Endosomal Sorting Complex Required for Transport) that assist in budding, and Z protein myristoylation at the conserved glycine residue at position 2 (G2), were shown to be important for Z-mediated budding [52,53,54,55,56]. These findings, along with earlier demonstration that, after chemical crosslinking, Z and N proteins form a complex in purified LCMV particles [51], and that LASV Z and N proteins interact with each other when overexpressed in mammalian cells [57], prompted the idea that Z-N interaction might be implicated in virion assembly. 

In order to investigate the contribution of viral protein-protein interactions to NW arenavirus morphogenesis, we developed a reverse genetic system able to drive TCRV-like nucleocapsids packaging along with Z and GP from JUNV into infectious chimeric virus-like particles (VLPs) [58]. As reported for OW arenavirus Zs, our studies indicated that upon solitary expression in mammalian cells, JUNV Z displays the ability to drive assembly and budding of Z-containing VLPs (self-budding activity), and that JUNV Z-mediated VLP production requires both the amino acid G2, and the integrity of the PTAP Late motif. Moreover, by dissecting the VLP system, we showed that JUNV Z is sufficient to recruit TCRV N protein into Z-induced particles that are surrounded by a lipid envelope, supporting the notion that Z-N interaction could facilitate the incorporation of nucleocapsids into budding particles [58]. 

Functional analysis of JUNV Z mutants in the VLP system led us to demonstrate that an intact RING structure and amino acid L79, which is strictly conserved across arenaviruses, are molecular determinants essential for JUNV Z to direct infectious chimeric VLP formation. These elements are also critical for the recruitment of N into Z-containing VLPs, whereas they are unnecessary for Z self-budding activity. Furthermore, both the L79 residue and the RING structure are required for intracellular Z-N interaction to occur. These results indicated that Z-N interactions are involved in infectious chimeric VLP assembly, strongly supporting that Z-N binding may contribute to the localization of viral nucleocapsids to the sites of budding at the plasma membrane and their subsequent packaging into arenavirus particles [58]. Further mutagenesis studies and VLP assays showed that highly conserved residues located in the vicinity of L79 outside the RING domain of JUNV Z protein (T81 and I83), may also participate in Z-N interaction [59]. In agreement, both amino acid L71 (equivalent to L79 in JUNV Z) and its neighboring residues in the C-terminal domain of LASV Z protein were shown to be critical for VLP infectivity, thus representing potential contacts with the viral nucleocapsid [60]. 

TCRV Z protein displays *bona fide* budding activity, despite containing a non-canonical (ASAP) Late domain [61]. The ability of TCRV Z to direct VLP production was reported to be enhanced upon coexpression with TCRV N protein [62], yet the underlying mechanism is unknown and no evidence of this effect has been described for any other arenavirus. As shown for JUNV Z, TCRV Z can also drive the incorporation of TCRV N into VLPs, in the absence of other viral proteins. A highly conserved YLCL motif located within the Z RING domain (Figure 1A), was found to be involved in TCRV N incorporation into TCRV Z-directed VLPs [62]. Similar results were observed for the OW Mopeia virus Z protein, whose YLCL motif was implicated in the interaction with the ESCRT-associated ALIX/AIP1 protein, proposed to link N and Z proteins during assembly [63]. Remarkably, the YLCL motif is also essential for TCRV Z to bind L [32], suggesting that Z-N and Z-L associations may be mutually exclusive.

Efforts to further characterize the N-Z interaction led our group to identify the C-terminal domain of TCRV N protein, spanning residues 360 to 570 (Figure 1C), as being required for the N-Z interaction and the recruitment of N into Z-induced VLPs [43]. Both the integrity of a conserved zinc binding motif and its flanking sequences were essential for this domain to be functional [43]. The involvement of the C-terminal half of N in its interaction with Z has also been reported for LCMV and Mopeia virus [64,65]. In the case of the latter, a region within this domain was also implicated in the interaction with the ALIX/AIP1 protein [63]. The observation that LCMV N and LASV Z cross-interacted, whereas LASV N and LCMV Z did not, suggests that species-specific residues -yet undetermined- within the C-terminal domain of N contribute to Z binding [64]. Overall, these results support the idea that the Z-N interaction -either direct or mediated by cellular protein(s)- plays an important role in viral assembly by promoting the plasma membrane targeting of the viral nucleocapsids.

### 3.2. GP-Z interaction

The arenavirus GPC is expressed as a single precursor polypeptide that is cleaved by cellular proteases to generate three subunits: the receptor-binding G1 protein, the transmembrane fusion subunit (G2) and a stable signal peptide SSP. Both the peripheral G1 and the SSP remain non-covalently associated with G2 forming the mature glycoprotein complex (GP), which assembles into trimers constituting the spikes on the virion envelope (reviewed in [66]). 

Binding to the matrix protein has been implicated in the recruitment of glycoprotein spikes into particles of other enveloped viruses [67,68]. Similarly, current evidence suggests that the incorporation of viral glycoproteins during arenavirus assembly requires interaction (either direct or indirect) with Z. Indeed, experimental data showed intracellular association between the Z and GP proteins from either LCMV or LASV, and that this interaction requires Z protein myristoylation [69], which is known to facilitate Z binding to cellular membranes [55,56]. In addition, pulldown experiments demonstrated that LASV GP and Z proteins interact with each other within VLPs released from GP- and Z-expressing cells [70]. In the case of JUNV proteins, evidence of GP incorporation into Z-induced VLPs has also been documented, further supporting the relevance of Z-GP association for GP assembly into viral particles [62]. 

Results from our laboratory using the chimeric TCRV/JUNV VLP system showed that coexpression of TCRV N was associated with a significant increment of G1 protein levels detected into VLPs, either in the presence or the absence of TCRV minigenome and L protein [58]. However, no apparent GP increase was observed into Z-directed VLPs when proteins corresponded entirely to JUNV [62]. Altogether, these observations suggest a role of Z-N complexes either in the efficient incorporation of GP or in stabilizing the GP structure on the particle envelope, specific to the chimeric system. Of note, the interaction between the matrix protein and the cytoplasmic domain of GP41 has been implicated in the stability of GP120-GP41 association on the surface of HIV particles [71]. 

### 3.3. Z oligomerization

Self-interaction of matrix proteins is thought to be critical for their function in budding of other enveloped viruses, such as Ebola and Rabies [72,73]. In the case of arenaviruses, evidence of self-interaction was initially provided for purified LCMV and LASV Z proteins expressed in bacteria [74]. Further studies of our laboratory to analyze the functional conformation of Z protein, conducted in mammalian cells, indicated that both TCRV and JUNV Z proteins self-associate into a ladder of oligomers, the putative dimer representing the major oligomeric form [32]. Analysis of a panel of Z mutants revealed that neither the integrity of the RING structure nor key residues required for Z-L contacts are involved in Z-Z interaction, indicating that homo-oligomerization is not needed for L recognition. Moreover, we demonstrated that amino acid G2, which is the N-terminal acceptor residue for protein myristoylation and conserved across the *Arenaviridae*, is required for self-association of TCRV and JUNV Z proteins [32]. The fact that residue G2 is also necessary for association of both Z proteins to the plasma membrane, strongly suggests that Z oligomerization is dependent on protein myristoylation and cell membrane targeting [32]. Whether myristate-promoted membrane association triggers Z homo-oligomerization hence stabilizing membrane binding, or alternatively, association of Z with the plasma membrane enhances Z local concentration facilitating its multimerization, is presently unknown. In any case, experimental data suggest that Z homo-oligomerization, which can be expected to present similar requirements for other arenaviruses, may play a critical role in the interaction between Z and GP and may represent a crucial step for virus assembly and budding.

Interestingly, we observed that upon coexpression in mammalian cells, the oligomerization-defective JUNV Z G2A mutant still displayed intracellular colocalization with N (Levingston, unpublished), suggesting that Z homo-oligomerization and plasma membrane targeting may not be required for its association with the N protein.

## 4. Regulation of arenavirus replication and particle assembly.

**Figure 2:**
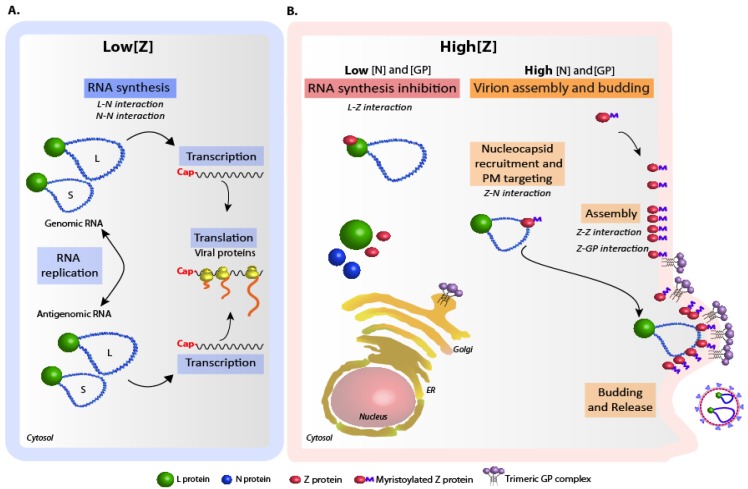
**Model of arenavirus replication and particle assembly regulation. **The relevant viral protein-protein interactions involved in each step are indicated. **(A) **Low intracellular levels of Z allow ongoing viral RNA synthesis. N-N and L-N interactions would operate during this process. **(B) **With concentrations of N and GP below a critical threshold, intracellular accumulation of Z results in polymerase activity inhibition through direct Z-L binding (left). When intracellular concentrations of N and GP are above the critical threshold, Z is engaged into virion assembly, and Z-mediated polymerase activity inhibition is prevented (right). In the last scenario, Z-N interaction mediates nucleocapsid recruitment and targeting to the plasma membrane (PM), where Z oligomerization and Z-GP interaction occur. Budding of the assembled particles is completed with the assistance of cellular proteins [77].

A basic question that remains unanswered is how the inhibitory activity of Z and its role in assembly are regulated. While low intracellular levels of Z allow ongoing viral RNA synthesis (Figure 2A), inhibition of polymerase activity may occur at high intracellular concentrations of Z [26,28,30,31,75]. In addition, engagement of Z in virus particle assembly through its interaction with other viral proteins (discussed above) might play a role in modulating Z inhibitory function. In this regard, a striking observation is that coexpression of GP significantly diminished Z-mediated inhibition of viral RNA synthesis both in the LCMV- and the chimeric TCRV/JUNV replicon systems [58,69]. Moreover, we found that in the presence of high intracellular GP levels, increasing amounts of N correlated with increasing levels of alleviation of Z-mediated inhibition of viral RNA synthesis in the context of TCRV components, suggesting that involvement of Z in interactions with N and GP would reduce the level of Z available to interact with L (Casabona, unpublished results). Thus, based on experimental data, a model can be proposed (Figure 2B) whereby intracellular accumulation of N and GP over a critical threshold might turn Z to be mainly engaged in protein-protein interactions leading to virion assembly. Alternatively, at concentrations of N and GP below such threshold, Z could be committed to viral RNA synthesis inhibition. Thus, Z functions might be temporally modulated according to the relative intracellular levels of viral proteins. Finally, it is possible that yet-unknown Z post-translational modifications -other than myristoylation- and/or the interplay of Z with cellular proteins could be involved in Z activities regulation. 

## 5. Concluding remarks

Studies on the interactions between viral proteins have improved our understanding of the mechanism of arenavirus genome expression and regulation, yet our knowledge of the details of these interactions remains limited. N homotypic interactions likely play a major role in RNA encapsidation, and may be required during viral RNA synthesis, when the interaction between N and L is assumed to operate. Further insight into the mechanism underlying these processes will be gained as more information of the structure of arenavirus nucleocapsids becomes available, as well as through additional studies using cell-based and *in vitro* reconstituted systems recreating viral RNA synthesis.

Current evidence supports the notion that Z protein operates as a key modulator of viral RNA synthesis by directly interacting with the L polymerase. However, little is known about the biological relevance of this Z function in the context of arenaviruses infections. Generation of recombinant viruses through reverse genetics, a methodology that has been developed for a number of arenaviruses [78,79,80,81,82], may provide the means for analyzing the role of this negative regulatory Z function in the context of acute and persistent viral infections. Other important questions, concerning the subcellular location of possible specialized sites of viral nucleocapsids recruitment by Z, how these complexes are eventually transported to the cell membrane, and the role of Z homo-oligomerization in Z-GP interaction, remain to be clarified. Further research on cellular factor(s) that could be involved in trafficking and assembly of the viral nucleocapsids, as well as in the Z-GP interaction is warranted. Future studies on viral protein-protein interactions relevant for viral gene expression, regulation and assembly, including those addressed to fully identify specific residues involved in diverse viral protein-protein complexes interfases, may provide important information on possible targets for the rational design of novel antiviral therapies against pathogenic arenaviruses.
